# Reliability of a human pose tracking algorithm for measuring upper limb joints: comparison with photography-based goniometry

**DOI:** 10.1186/s12891-022-05826-4

**Published:** 2022-09-21

**Authors:** Jingyuan Fan, Fanbin Gu, Lulu Lv, Zhejin Zhang, Changbing Zhu, Jian Qi, Honggang Wang, Xiaolin Liu, Jiantao Yang, Qingtang Zhu

**Affiliations:** 1 Department of Microsurgery, Orthopedic Trauma and Hand Surgery, The First Affiliated Hospital, Sun Yat-Sen University, Guangzhou, 510080 China; 2 Guangdong AICH Technology Co.Ltd, Guangzhou, 510080 China; 3 Guangdong Province Engineering Laboratory for Soft Tissue Biofabrication, Sun-Yat-Sen University, Guangzhou, 510080 China; 4 Guangdong Provincial Key Laboratory for Orthopedics and Traumatology, Guangzhou, 510080 China

**Keywords:** Pose estimation, Range of motion, Goniometry, Automatic measurement, Photography, Reliability

## Abstract

**Background:**

Range of motion (ROM) measurements are essential for diagnosing and evaluating upper extremity conditions. Clinical goniometry is the most commonly used methods but it is time-consuming and skill-demanding. Recent advances in human tracking algorithm suggest potential for automatic angle measuring from RGB images. It provides an attractive alternative for at-distance measuring. However, the reliability of this method has not been fully established. The purpose of this study is to evaluate if the results of algorithm are as reliable as human raters in upper limb movements.

**Methods:**

Thirty healthy young adults (20 males, 10 females) participated in this study. Participants were asked to performed a 6-motion task including movement of shoulder, elbow and wrist. Images of movements were captured by commercial digital cameras. Each movement was measured by a pose tracking algorithm (OpenPose) and compared with the surgeon-measurement results. The mean differences between the two measurements were compared. Pearson correlation coefficients were used to determine the relationship. Reliability was investigated by the intra-class correlation coefficients.

**Results:**

Comparing this algorithm-based method with manual measurement, the mean differences were less than 3 degrees in 5 motions (shoulder abduction: 0.51; shoulder elevation: 2.87; elbow flexion:0.38; elbow extension:0.65; wrist extension: 0.78) except wrist flexion. All the intra-class correlation coefficients were larger than 0.60. The Pearson coefficients also showed high correlations between the two measurements (*p* < 0.001).

**Conclusions:**

Our results indicated that pose estimation is a reliable method to measure the shoulder and elbow angles, supporting RGB images for measuring joint ROM. Our results presented the possibility that patients can assess their ROM by photos taken by a digital camera.

**Trial registration:**

This study was registered in the Clinical Trials Center of The First Affiliated Hospital, Sun Yat-sen University (2021–387).

**Supplementary Information:**

The online version contains supplementary material available at 10.1186/s12891-022-05826-4.

## Background

Joint range of motion (ROM) is a measure of interest in clinical practice as it is significant for the diagnosis, functional assessment and treatment evaluation of the upper extremity. It is reported that measurement of ROM is required in more than 80% of commonly used function assessment scales for the shoulder and elbow [1]. Conventionally, the measurement of ROM was performed by manual goniometry [2]. The goniometer is low-cost and portable, but its reliability highly depends on the rater's experience [3]. Moreover, the procedure is demanding and time-consuming, which may impact the efficiency of medical care.

In addition, with the rapid development of telemedicine, how to determine the joint movement at-distance has peaked the interests of many researches [4–9]. Typical motion capture system could provide accurate kinematics measurement [10, 11] but requires large space for data collection, which makes it costly, not portable, and thus impractical for home-use. Advances in smartphone technology, specifically the build-in sensors and high-resolution cameras, provides a potential platform for joint measurement. The number of mobile application used for clinical assessment have considerably increased in recent years [12]. There are two main groups of these applications using the embedded inclinometer and images taken by phone camera. Mitchell et al. had evaluated the reliability of two applications, one from each group, in the measurement of shoulder rotation, and indicated that both of the methods had acceptable reliability compared with standard goniometry [13]. Subsequent studies also confirmed this finding and the mean difference between the two methods and manual goniometry ranged from 0.2 to 6.4 (inclinometer-based) [5–7, 14] and 0.1 to 11.9 (photographic-based) [15, 16]. According to previous studies, the inclinometer-based method could provide more consistent results and detect slighter changes [17, 18]. Morey et al. have reported the minimal detectable change of digital inclinometer in shoulder measurement ranging from 4 to 9 degrees [17]. However, the measuring process of this method was relatively complicated. The participants need to attach the instrument to specific positions [5, 7, 18] and changing the position could lead to measurement errors [17]. Compared with the inclinometer-based method, the photographic-based method provides easier procedure to follow and contactless measuring process [8]. In addition, it is possible for doctors to know whether the measurement was correctly performed through the photos, which is extremely important for patient self-assessment. However, the existing application still need raters to mark on the photos [19], which means it could not actually reduce the workload of therapist nor the subjectivity of results.

Therefore, an object, accurate and automatic method is desired. Recent advances in human tracking algorithm offers a new option for this task. This kind of algorithm can detect the coordinate of a set of joint points from images. Through the position of these points (shoulder, elbow, etc.,), the pose of person can be described and the angle of joints could also be calculated, which provides an attractive alternative for at-distance measuring [11, 20]. In this study, we employed OpenPose, one of the most widely used method proposed by Cao et al. [21] to estimate joint position from RGB images. Previous articles have evaluated the reliability of OpenPose-based system in gait analysis [22] and Parkinson rating [23, 24]. Ota et al. also compared OpenPose and VICON (a 3D motion capture system) in measuring lower limb joint angle and found significant associations of the two methods [11]. However, the utility of OpenPose in the assessment of upper limb angle remains unclear. Herein we constructed a measuring setup based on this algorithm, using RGB images to measure upper limb movements. This study evaluates the reliability of OpenPose for clinical measurement by comparing the results with photography-based goniometry.

## Materials and methods

### Participants

Thirty healthy young adults (20 males, 10 females, 22–35 years old), with no claim of medical history nor impairment in the upper limbs participated in this study. This study was approved by the institutional review board of our institution (2021–387). Estimated sample size was calculated by PASS software (version 15.0) using equivalence test for the difference between two means. With a type one error (α) of 0.05, power (1-β) of 0.95, equivalence limit of 10 degree, and standard deviation of 10, that a minimum of 27 samples would be required. All subjects were given full explanations about the motion tasks. After that, written consent was obtained for the use of their images for research purposes.

### Measurement setup

Since many factors such as the distance to cameras, the angle and height of cameras could affect the measurement results, we constructed a standardized measurement environment in this study, shown in Supplementary Fig. 1A. Three commercial digital cameras with 2560 × 1920 resolution and 79 degrees field of view (HIKIVISION DS-2CD3T56FWDV2-I3) were positioned around the field (one in the front and two in the sides). The height of the cameras was 1.5 m and the distance between the camera and subjects was 3 m. To ensure the consistency of the participant placement, feet markers were placed 3 m away from the cameras. The environment was illuminated by normal white light from LED sources. The background was a white wall without decoration.

### Motion tasks and parameters extracting

We designed a 6-task procedure including shoulder abduction, shoulder elevation, elbow flexion, elbow extension, wrist flexion and extension (Shown in Supplementary Fig. 1B). All the motion tasks were completed in the above mentioned environment. To control the impact introduced by rotation, all the interest angles in our design were fully presented in either sagittal or coronal view. Participants were asked to stand in the field and perform the motion tasks one after another. To ensure their performances were the same as we recommended, we set a screen in front of participants with word and video instructions. Moreover, their motion videos were real-time displayed on that screen as well. All photographs were taken from the anterior side, except the elbow flexion was taken from the lateral side (one for each side).

### Automatic measurement

The landmarks of each joint were estimated by the Openpose Human Pose Estimation library (version 1.5.0) [21]. The coordinates for landmarks of joints were further extracted, and skeleton models were rebuilding accordingly. Then, the joint angle was calculated by corresponding coordinates using the following formula.

$$\theta =\mathrm{arccos}\left(\frac{\overrightarrow{a}\bullet \overrightarrow{b}}{\left|\overrightarrow{a}\right|\bullet \left|\overrightarrow{b}\right|}\right)$$

### Digital photography-based measurement

After the automatic measurement, the photography-based measurements were conducted by using the same images. The angle of joints was measured by two hand surgeons individually, applying a screen goniometer software to the images displayed on the computer screen (The main reason of screen-goniometry was to make sure the posture present to measurement system and human researchers were identical. The validity of this method have been previously confirmed [25, 26]). To minimize the uncertainty of manual assessment, these images were reassessed by the same researchers at an interval of one week. The landmarks included the center of the shoulder, elbow and wrist, axis along the center of the upper arm and forearm, and central axis along the metacarpals. During the measurement, surgeons were free to locate the landmarks after reading the instruction. During this procedure, observers were not allowed to see the results of automatic measurement or another observer's report.

### Data processing and statistical analysis

The mean values of the four measurements (2 researchers * 2 round) were considered as the standard results for comparison. All measurements are presented as mean ± standard deviation (means ± sd). The deviation between the automatic assessment and standard results and the 95% confidence interval (CI) were calculated to assess the accuracy. The intra-class correlation coefficient (ICC) was also performed between the standard and the proposed measurement for assessing the agreement. Next, the results were analyzed using Bland and Altman analysis [27]. The upper limits of agreement (LOA) were considered reference values to judge if the proposed measurement could be a reliable method for upper limb ROM. As the results of Openpose, like other deep learning models, were calculated by a series of formulas, it is not hard to conclude that the results would be in complete agreement when analyzing the same image twice. So the repeatability of the automatic methods was not assessed. In comparison, the repeatability of manual measurement was evaluated by comparing the test–retest results. In addition, to confirm the reliability, linear regression analyses were conducted to compare the manual and system measurement data. R-square was calculated to evaluate the correlation between different methods.

Statistical analysis was performed by SPSS 22.0 (Armonk, NY: IBM Corp) and R software 4.0.3 (R Foundation for Statistical Computing, Vienna, Austria). Results with *p* < 0.05 were considered statistically significant. Interpretation of ICC value was as follows: < 0.20: unacceptable, 0.20–0.40: questionable, 0.41–0.60: good, 0.61–0.80 very good, 0.81–1.00: excellent. The correlation coefficient, 1 indicates a total positive linear correlation, 0 means no linear correlation, and -1 shows a total negative linear correlation.

## Results

**Fig. 1:**
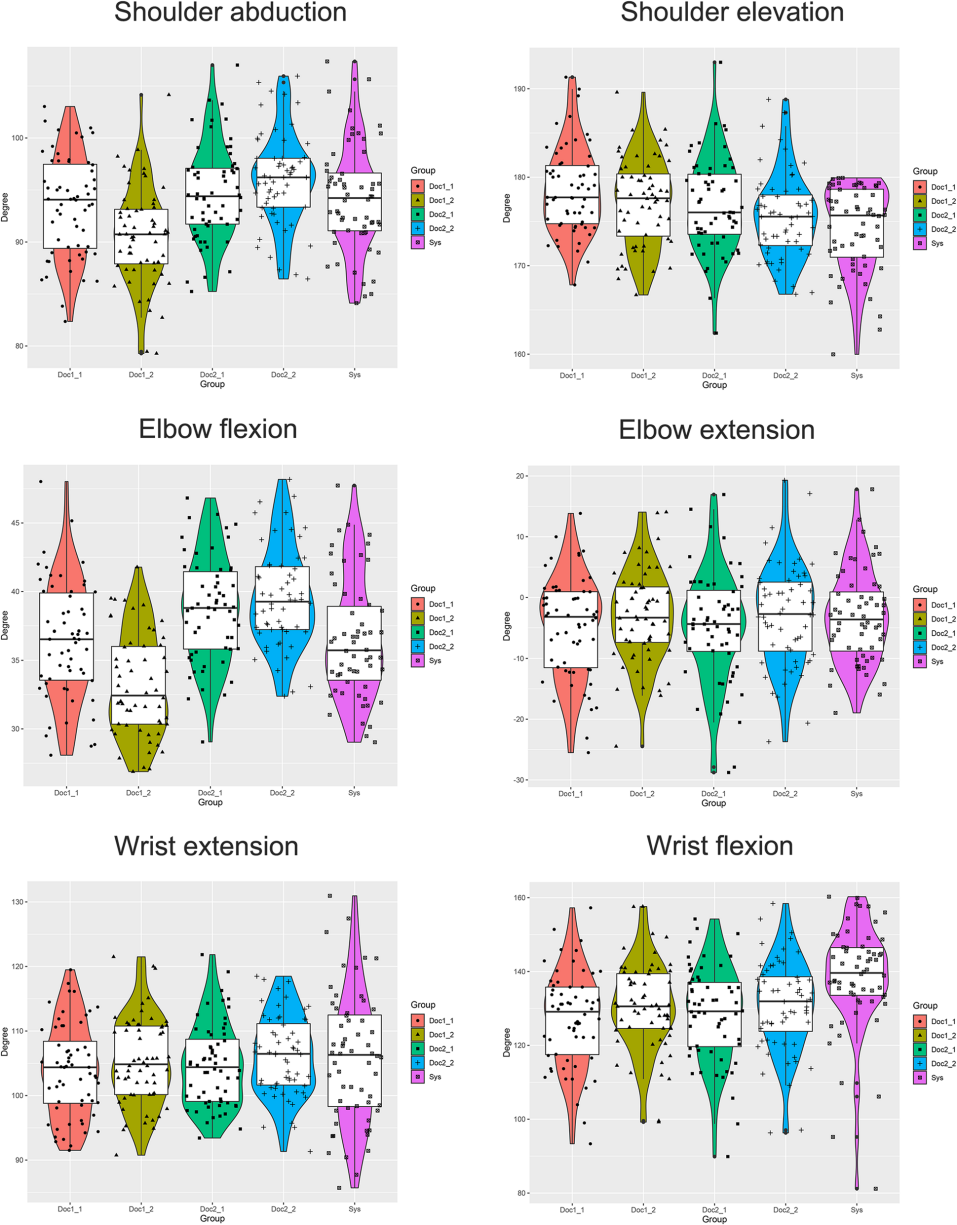
Comparison of the measurement results of the 6 motions. Sys: The automatic measuring system; Doc1_1: The first measurement of the first doctor, the rest are in the same manner

The measuring results in the shoulder, elbow and wrist measured by two observers and the human tracking algorithm are summarized in Table 1 and Fig. 1. The example of automatic measurements result is shown in Supplementary Fig. 2.

**Table 1:** Summary of the measurement results of different methods

Motion Tasks	Sys $\left(\overline{\mathrm{x}}\pm \mathrm{sd}\right)$ (degree)	Doc1 $\left(\overline{\mathrm{x}}\pm \mathrm{sd}\right)$ (degree)	Doc2 $\left(\overline{\mathrm{x}}\pm \mathrm{sd}\right)$ (degree)	Doc_mean $\left(\overline{\mathrm{x}}\pm \mathrm{sd}\right)$ (degree)
Shoulder abduction	94.2 ± 5.13	92 ± 4.93	95.38 ± 4.5	93.69 ± 5
Shoulder elevation	174.15 ± 4.82	177.86 ± 4.91	176.17 ± 5.08	177.02 ± 5.06
Elbow flexion	36.47 ± 4.33	34.77 ± 4.29	39.1 ± 3.73	36.94 ± 4.56
Elbow extension	-3.21 ± 7.51	-3.91 ± 7.92	-3.82 ± 8.73	-3.87 ± 8.32
Wrist extension	105.84 ± 10.22	104.63 ± 6.92	105.4 ± 6.3	105.02 ± 6.62
Wrist flexion	138.28 ± 14.57	128.95 ± 12.44	129.68 ± 12.67	129.32 ± 12.54

*Sys* The automatic measuring system, *Doc* Doctor, *sd* Standard deviation

### Pose estimation

The poses of participants were successfully estimated in all but two images, and both were because of the person detection failure (The reason of error was due to these pictures included more than one person and the angle calculation was performed on the wrong target). The success rate was 99.44% (358/360).

### Difference between observers

The results of the inter and intra-observer comparison are presented in Tables 2 and 3. There was excellent agreement between observers, with mean difference ranging from 0.08 to 4.33 and ICC value ranging from 0.897 to 0.951. The intra-observer comparison also indicates a good consistency, the mean differences between test and re-test measurements were less than 5 degrees.

**Table 2:** Summary of the inter-group differences

Motion Tasks	Sys vs Doc_mean (degree)		Doc1_1 vs Doc1_2 (degree)		Doc2_1 vs Doc2_2 (degree)		Doc1 vs Doc2 (degree)	
	$\overline{\mathrm{x}}\pm \mathrm{sd}$	95%CI	$\overline{\mathrm{x}}\pm \mathrm{sd}$	95%CI	$\overline{\mathrm{x}}\pm \mathrm{sd}$	95%CI	$\overline{\mathrm{x}}\pm \mathrm{sd}$	95%CI
Shoulder abduction	-0.51 ± 4.38	(-1.64–0.62)	2.93 ± 4.83	(1.68–4.18)	-1.35 ± 4.61	(-2.54–0.16)	-3.38 ± 2.08	(-3.92–2.85)
Shoulder elevation	2.87 ± 4.84	(1.62–4.11)	1.35 ± 4.35	(0.23–2.48)	1.22 ± 3.99	(0.19–2.26)	1.68 ± 2.16	(1.13–2.24)
Elbow flexion	0.38 ± 3.27	(-0.33–1.09)	3.28 ± 4.91	(2.00–4.58)	-0.85 ± 4.38	(-2.00–0.30)	-4.33 ± 1.88	(-4.82–3.84)
Elbow extension	-0.65 ± 5.48	(-2.07–0.76)	-1.76 ± 8.25	(-3.89–0.38)	-1.84 ± 8.92	(-4.14–0.47)	-0.08 ± 3.57	(-1.01–0.84)
Wrist extension	-0.78 ± 8.40	(-0.55–1.39)	-1.47 ± 8.29	(-3.62–0.67)	-1.46 ± 7.11	(-3.29–0.38)	-0.77 ± 2.61	(-1.44–0.09)
Wrist flexion	-8.96 ± 12.71	(-12.24–5.68)	-3.33 ± 16.70	(-7.64–0.99)	-2.24 ± 16.86	(-6.60–2.11)	-0.73 ± 4.01	(-1.77–0.30)

*Sys* The automatic measuring system, *Doc1_1* The first measurement of the first doctor, the rest are in the same manner, *Doc_mean* The average measuring value of doctors, *sd* Standard deviation, *CI* Confidence interval

**Table 3:** Summary of the intra-class correlation coefficients

Motion Tasks	Sys vs. Doc_mean		Doc1 vs.Doc2	
	ICC	95%CI	ICC	95%CI
Shoulder abduction	0.693	0.485–0.816	0.925	0.875–0.955
Shoulder elevation	0.620	0.363–0.773	0.940	0.899–0.964
Elbow flexion	0.787	0.639–0.874	0.897	0.827–0.939
Elbow extension	0.831	0.717–0.899	0.933	0.888–0.960
Wrist extension	0.620	0.363–0.773	0.937	0.894–0.962
Wrist flexion	0.623	0.369–0.775	0.951	0.919–0.971

*Sys* The automatic measuring system, *Doc_mean* The average measuring value of doctors

### Difference between observer and machine

**Fig. 2:**
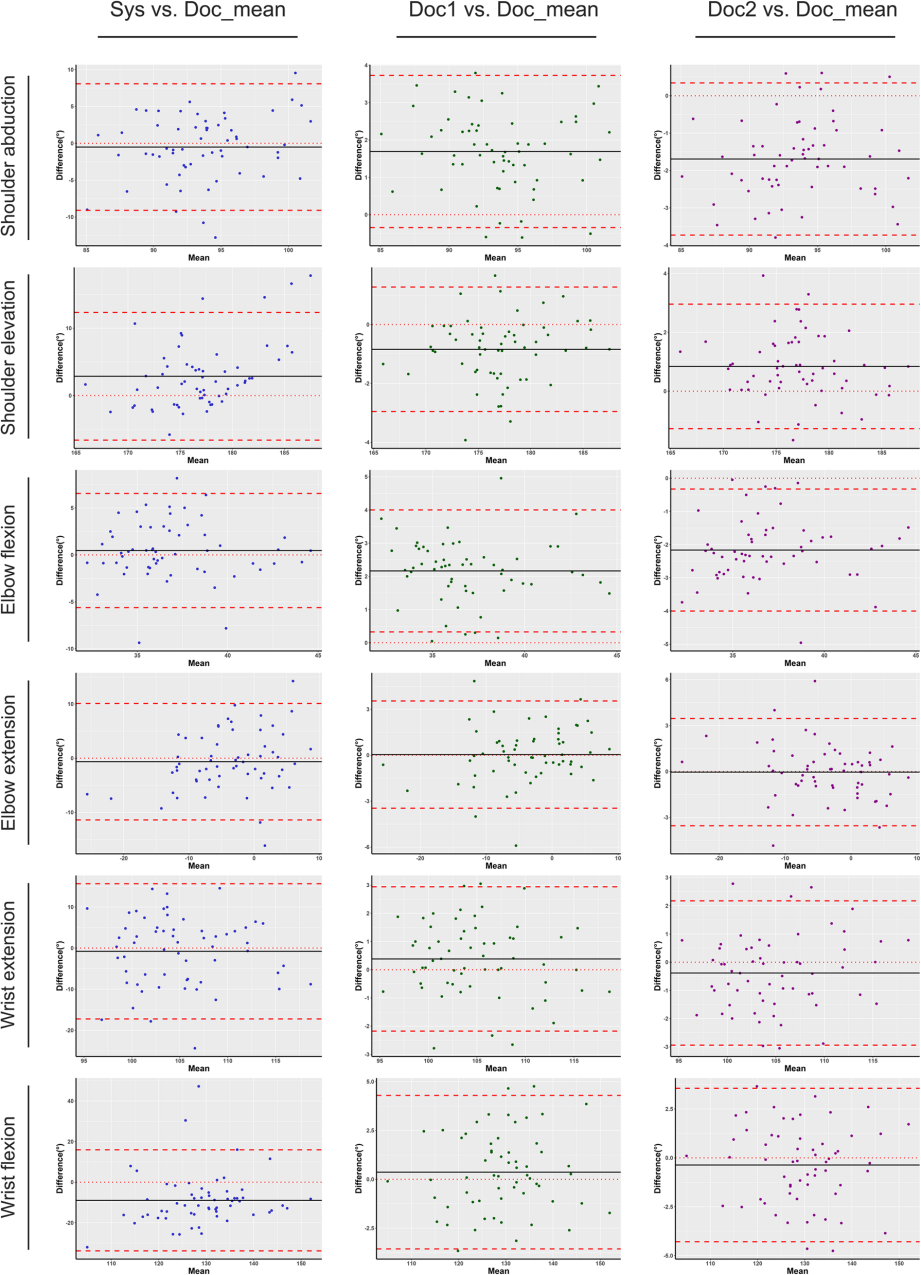
Bland–Altman plots for inter-rater agreement. Sys: The automatic measuring system; Doc: Doctor; Doc_mean: The average measuring value of doctors; This plot compares the individual measurement result with the average value of doctors. The x-axis represents the mean value; the y axis represents the inter-rater difference. The dotted lines represent the limit of differences

**Fig. 3:**
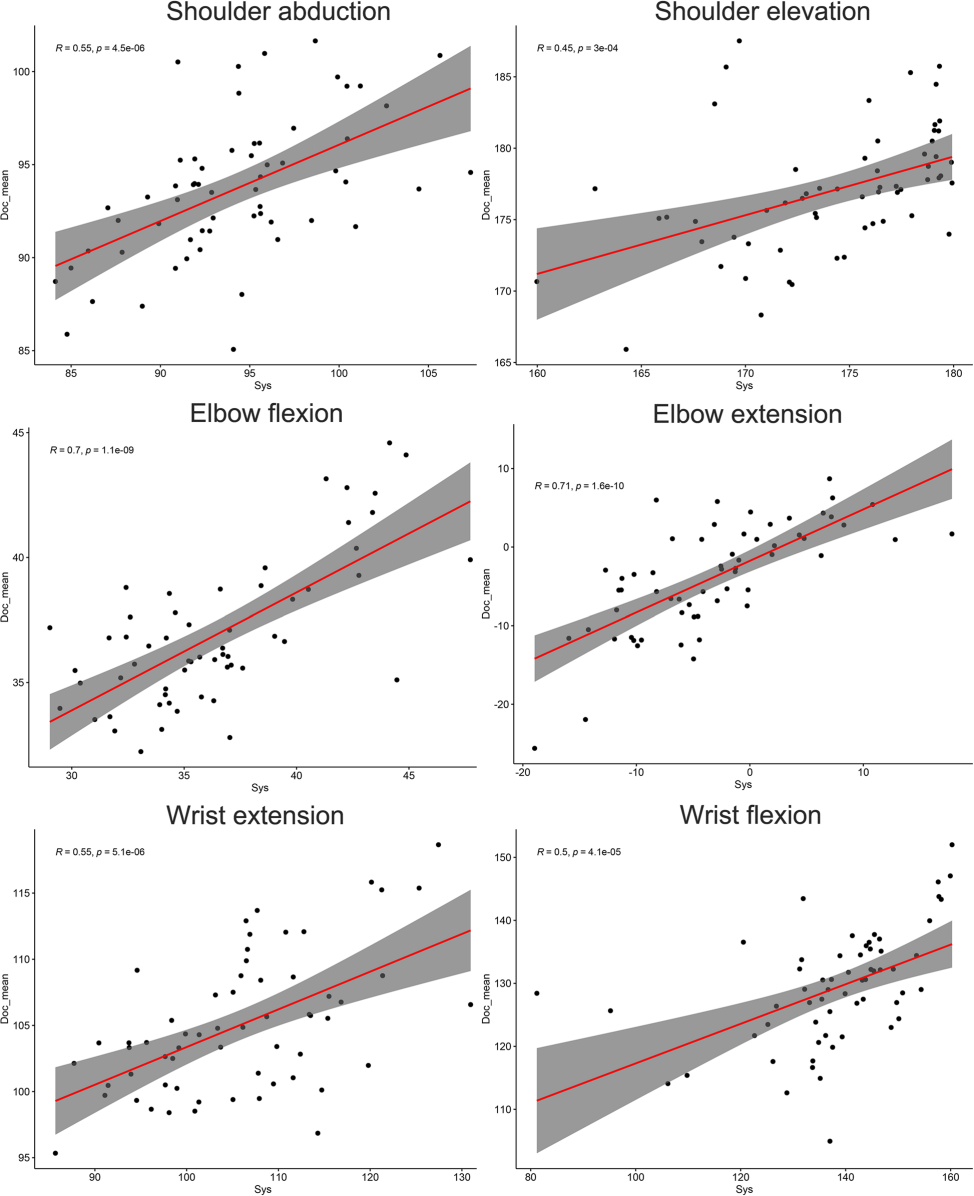
The linear correlation between raters. Sys: The automatic measuring system; Doc_mean: The average measuring value of doctors

As shown in Table 2, the observer-system differences were comparable to the inter and intra-observer difference. The most significant difference was found in wrist flexion (8.96 ± 12.71; 95%CI: -12.24–5.68). In the other 5 motions, the 95% confidence intervals of the mean differences between manual and automatic assessment were less than 5 degrees. Similarly, the Bland–Altman plots also indicate acceptable agreements for the shoulder and elbow motions. In comparison, the conformity for wrist motions is relatively poor (Fig. 2), as the credible intervals were more than 10 degrees. Then, the consistency was further evaluated by ICC values. The results suggested a good to excellent agreement (ICC > 0.60) in all motions (Table 3). The lowest consistency was found in shoulder elevation and wrist extension (ICC = 0.620), while the best was found in elbow extension (ICC = 0.831). Additionally, linear correlations between system and observer measurement were also demonstrated (R ranges from 0.45 to 0.71, *p* < 0.001 Fig. 3).

## Discussion

The range of motion (ROM) of the upper limb is an important clinical parameter to various functional evaluations before and after treatment. Conventionally, ROM was assessed manually using the standard goniometer. This procedure is time-consuming and requires expertise. In addition, various reasons such as financial and geographic factors, and busy schedule could prevent patients from clinic visiting [28]. Therefore, telemedicine has become popular as a method of patient evaluation. Photographs are easily obtained and disseminated in our daily life. Getting movement parameters from remote photographs has potential to decrease the cost of physical evaluation. Human pose tracking algorithms can automatically calculate joint angles from RGB images and provide a new option for the remote evaluation. However, the reliability of this method is extremely important before the using in clinical settings.

This study sought to evaluated the reliability of an automatic goniometry method. The testing environments in this study is also possible to be set up in patient's home. In our analysis, we found that the algorithm-based method has acceptable reliability compared to human observers. The results indicate that the differences between the proposed method and the average value of observers are less than 5 degrees in shoulder and elbow motions, comparable to the inter and intra-observer differences. Compared to that reported in previous studies, these differences are notably more minor than that of visual estimation [15, 29] and are comparable to inertial sensors [30] and depth camera [31]. Therefore, the proposed method may have great accuracy and reliability in measuring ROMs of the shoulder and elbow.

In this study, the greatest observer-machine difference was found in wrist flexion, and the mean value was 8.96 degrees. However, this reliability is still competitive compared to other image-based applications [6, 32]. Nevertheless, as seen in the Bland–Altman plots, we found the angle was over-estimated by the system in most cases. Thus, we speculate this might be a systematic error that could be correct when a larger sample size is available.

It is difficult for participants to keep their posture still during measuring, as previous studies indicated [33, 34]. According to the literature, several methods were employed to minimize this problem. Cook et al. used a wooden triangle with fixed internal angles to support the joints of interest during assessing [35]. In comparison, Chang et al. adopted a glass plate as hand support to reduce movement during the 3D scanning process [36]. More commonly, many studies choose the 3D motion capture system to achieve data collecting [37–39] simultaneously and thus minimize the differences caused by involuntary posture changing. Our study compared the results of the automatic system and human observers by measuring the same image individually. In this way, we can conclude the actual differences between the two methods without impacting the inconsistency of motions. The concept of obtaining joint ROM from photographs is not new. Previous studies have indicated that it is accurate and reliable compared with conventional clinical goniometry [25, 26]. Additionally, the results of image-based goniometry could be more consistent than that of the conventional way in some cases [26]. This present study also proved the value of screen goniometry as a reliable alternative for measuring, with slight inter and intra-observer differences.

There are still some limitations of our study: Firstly, although the comparison between the OpenPose algorithm and human observers revealed clinical reliability, future validity studies utilizing the motion capture system as a standard method are still needed to clarify the accuracy. Secondly, the participants were limited to young, healthy persons, and did not included the elderly nor the patients, making the results statistically less robust and lessening the generalizability of the proposed method. Next, motions with rotation were not assessed because it was hard to estimate 3D motions through 2D images. Although it could be an inevitable technical error, this issue will be the aim of our future studies. In addition, angle of joints may contain the movements of several joints (For example, the angle of shoulder joint includes the movement of the scapula, thorax, and thoracic spine) which lead to inaccurate of measurement, but we believe that is still good enough for telemedicine system. Another drawback is that the accuracy of our method depends on the compliance and cooperation of participants to some extent. If the subject cannot properly understand our purposes, the results can exhibit deviation.

## Conclusions

This study demonstrates a reliable method to measure joint ROM of the upper limb using RGB photographs. We have proved the reliability of the proposed method by comparing it with photography-based goniometry. Our results indicated that this human pose tracking algorithm could act as an exciting alternative to conventional goniometry. Its use may benefit the remote evaluation as users can obtain reliable kinematics parameters personally without traveling to clinical centers. However, it would be interesting to implement a study with a larger sample of patients or the elders with movement disorders and study more motions.

## Supplementary Information

**Additional file 1: Supplementary Figure 1.** Measurement setup during the 6-motion task. A) setups of the environment; B) diagram of the 6-motions task.

**Additional file 2: Supplementary Figure 2.** Results obtained from the proposed method.

## Data Availability

The data analyzed during this study are not publicly available due to containing identifying information (the accuracy of the algorithm would be impacted if the eyes/facial region was blurring) but are available from the corresponding author on reasonable request.

## Abbreviations

ROM: Range of motion
LOA: Limits of agreement
ICC: Intra-class correlation coefficient
CI: Confidence interval

## References

[1] Suk M, Hanson B, Norvell D, Helfet D (2009). Musculoskeletal Outcomes Measures and Instruments. Vol 1.

[2] Boone DC, Azen SP, Lin CM, Spence C, Baron C, Lee L (1978). Reliability of goniometric measurements. Phys Ther.

[3] Bovens AM, van Baak MA, Vrencken JG, Wijnen JA, Verstappen FT (1990). Variability and reliability of joint measurements. Am J Sports Med.

[4] Naeemabadi M, Dinesen B, Andersen OK, Madsen NK, Simonsen OH, Hansen J (2019). Developing a telerehabilitation programme for postoperative recovery from knee surgery: specifications and requirements. BMJ Health Care Inform.

[5] Behnoush B, Tavakoli N, Bazmi E, Nateghi Fard F, Pourgharib Shahi MH, Okazi A, Mokhtari T (2016). Smartphone and universal goniometer for measurement of elbow joint motions: a comparative study. Asian J Sports Med.

[6] Lendner N, Wells E, Lavi I, Kwok YY, Ho PC, Wollstein R (2019). Utility of the iPhone 4 Gyroscope Application in the Measurement of Wrist Motion. Hand (New York, NY).

[7] Vauclair F, Aljurayyan A, Abduljabbar FH, Barimani B, Goetti P, Houghton F, Harvey EJ, Rouleau DM (2018). The smartphone inclinometer: a new tool to determine elbow range of motion?. Eur J Orthop Surg Traumatol.

[8] Meislin MA, Wagner ER, Shin AY (2016). A comparison of elbow range of motion measurements: smartphone-based digital photography versus goniometric measurements. J Hand Surg Am.

[9] Buvik A, Bugge E, Knutsen G, Småbrekke A, Wilsgaard T (2016). Quality of care for remote orthopaedic consultations using telemedicine: a randomised controlled trial. BMC Health Serv Res.

[10] Kim SH, Kwon OY, Park KN, Jeon IC, Weon JH (2015). Lower extremity strength and the range of motion in relation to squat depth. J Hum Kinet.

[11] Ota M, Tateuchi H, Hashiguchi T, Kato T, Ogino Y, Yamagata M, Ichihashi N (2020). Verification of reliability and validity of motion analysis systems during bilateral squat using human pose tracking algorithm. Gait Posture.

[12] Buechi R, Faes L, Bachmann LM, Thiel MA, Bodmer NS, Schmid MK, Job O, Lienhard KR (2017). Evidence assessing the diagnostic performance of medical smartphone apps: a systematic review and exploratory meta-analysis. BMJ Open.

[13] Mitchell K, Gutierrez SB, Sutton S, Morton S, Morgenthaler A (2014). Reliability and validity of goniometric iPhone applications for the assessment of active shoulder external rotation. Physiother Theory Pract.

[14] Kim TS, Park DD, Lee YB, Han DG, Shim JS, Lee YJ, Kim PC (2014). A study on the measurement of wrist motion range using the iPhone 4 gyroscope application. Ann Plast Surg.

[15] Hayes K, Walton JR, Szomor ZR, Murrell GA (2001). Reliability of five methods for assessing shoulder range of motion. Aust J Physiother.

[16] Scott KL, Skotak CM, Renfree KJ (2019). Remote Assessment of wrist range of motion: inter- and intra-observer agreement of provider estimation and direct measurement with photographs and tracings. J Hand Surg.

[17] Kolber MJ, Vega F, Widmayer K, Cheng MS (2011). The reliability and minimal detectable change of shoulder mobility measurements using a digital inclinometer. Physiother Theory Pract.

[18] Boissy P, Diop-Fallou S, Lebel K, Bernier M, Balg F, Tousignant-Laflamme Y (2017). Trueness and minimal detectable change of smartphone inclinometer measurements of shoulder range of motion. Telemed J E Health.

[19] Ma M, Proffitt R, Skubic M (2018). Validation of a Kinect V2 based rehabilitation game. PLoS One.

[20] Zago M, Luzzago M, Marangoni T, De Cecco M, Tarabini M, Galli M (2020). 3D Tracking of human motion using visual skeletonization and stereoscopic vision. Front Bioeng Biotechnol.

[21] Cao Z, Hidalgo G, Simon T, Wei S-E, Sheikh Y (2019). OpenPose: realtime multi-person 2D pose estimation using Part Affinity Fields. IEEE Trans Pattern Anal Mach Intell.

[22] Ota M, Tateuchi H, Hashiguchi T, Ichihashi N (2021). Verification of validity of gait analysis systems during treadmill walking and running using human pose tracking algorithm. Gait Posture.

[23] Park KW, Lee EJ, Lee JS, Jeong J, Choi N, Jo S, Jung M, Do JY, Kang DW, Lee JG (2021). Machine learning-based automatic rating for cardinal symptoms of Parkinson disease. Neurology.

[24] Sato K, Nagashima Y, Mano T, Iwata A, Toda T (2019). Quantifying normal and parkinsonian gait features from home movies: Practical application of a deep learning-based 2D pose estimator. PLoS One.

[25] Armstrong AD, MacDermid JC, Chinchalkar S, Stevens RS, King GJ (1998). Reliability of range-of-motion measurement in the elbow and forearm. J Shoulder Elbow Surg.

[26] Blonna D, Zarkadas PC, Fitzsimmons JS, O'Driscoll SW (2012). Validation of a photography-based goniometry method for measuring joint range of motion. J Shoulder Elbow Surg.

[27] Bland JM, Altman DG (1986). Statistical methods for assessing agreement between two methods of clinical measurement. Lancet.

[28] Jacklin PB, Roberts JA, Wallace P, Haines A, Harrison R, Barber JA, Thompson SG, Lewis L, Currell R, Parker S (2003). Virtual outreach: economic evaluation of joint teleconsultations for patients referred by their general practitioner for a specialist opinion. BMJ.

[29] Terwee CB, de Winter AF, Scholten RJ, Jans MP, Devillé W, van Schaardenburg D, Bouter LM (2005). Interobserver reproducibility of the visual estimation of range of motion of the shoulder. Arch Phys Med Rehabil.

[30] Beshara P, Chen JF, Read AC, Lagadec P, Wang T, Walsh WR (2020). The reliability and validity of wearable inertial sensors coupled with the Microsoft Kinect to measure shoulder range-of-motion. Sensors (Basel, Switzerland).

[31] Lee SH, Yoon C, Chung SG, Kim HC, Kwak Y, Park HW, Kim K (2015). Measurement of shoulder range of motion in patients with adhesive capsulitis using a Kinect. PLoS One.

[32] Ienaga N, Fujita K, Koyama T, Sasaki T, Sugiura Y, Saito H (2020). Development and user evaluation of a smartphone-based system to assess range of motion of wrist joint. J Hand Surg Glob Online.

[33] Yu F, Zeng L, Pan D, Sui X, Tang J (2020). Evaluating the accuracy of hand models obtained from two 3D scanning techniques. Sci Rep.

[34] Zl A, Ccc B, Pgd B, Xc A (2008). Refraction effect analysis of using a hand-held laser scanner with glass support for 3D anthropometric measurement of the hand: strategy comparison and application. J Measurement.

[35] Cook JR, Baker NA, Cham R, Hale E, Redfern MS (2007). Measurements of wrist and finger postures: a comparison of goniometric and motion capture techniques. J Appl Biomech.

[36] Chang C-C, Li Z, Cai X, Dempsey P (2007). Error control and calibration in three-dimensional anthropometric measurement of the hand by laser scanning with glass support. J Measurement.

[37] Zulkarnain RF, Kim GY, Adikrishna A, Hong HP, Kim YJ, Jeon IH (2017). Digital data acquisition of shoulder range of motion and arm motion smoothness using Kinect v2. J Shoulder Elbow Surg.

[38] Wilson JD, Khan-Perez J, Marley D, Buttress S, Walton M, Li B, Roy B (2017). Can shoulder range of movement be measured accurately using the Microsoft Kinect sensor plus Medical Interactive Recovery Assistant (MIRA) software?. J Shoulder Elbow Surg.

[39] Chu H, Joo S, Kim J, Kim JK, Kim C, Seo J, Kang DG, Lee HS, Sung KK, Lee S (2018). Validity and reliability of POM-Checker in measuring shoulder range of motion: protocol for a single center comparative study. Medicine.

