# Supplementary figures and images for: Reliability of a human pose tracking algorithm for measuring upper limb joints: comparison with photography-based goniometry
Source: BMC Musculoskelet Disord. 2022 Sep 21;23:877. doi: 10.1186/s12891-022-05826-4 (PMC9490917; doi:10.1186/s12891-022-05826-4)

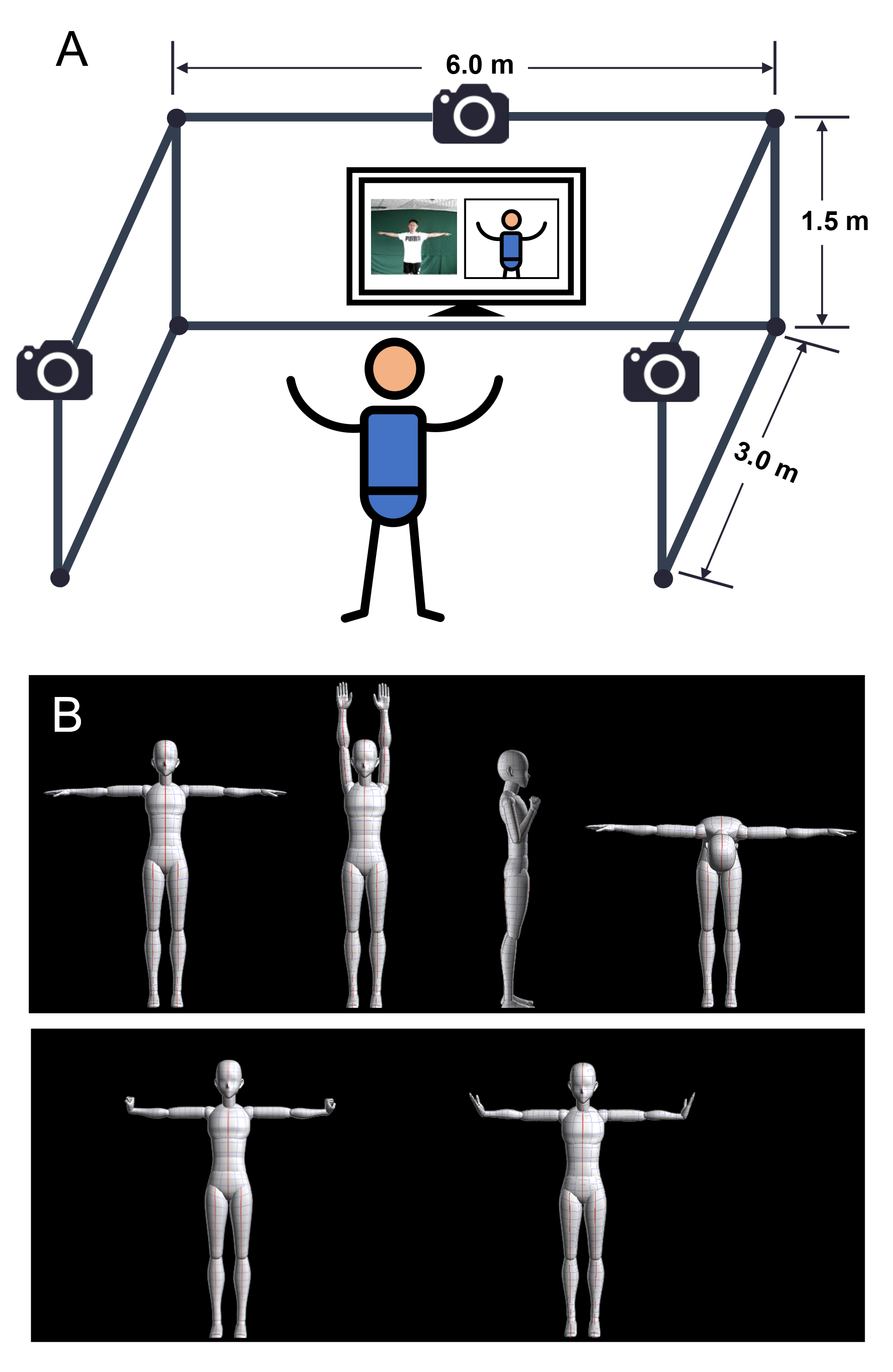

Supplement: Supplementary file 1 — Additional file 1: Supplementary Figure 1. Measurement setup during the 6-motion task. A) setups of the environment; B) diagram of the 6-motions task. [file 12891_2022_5826_MOESM1_ESM.tif]

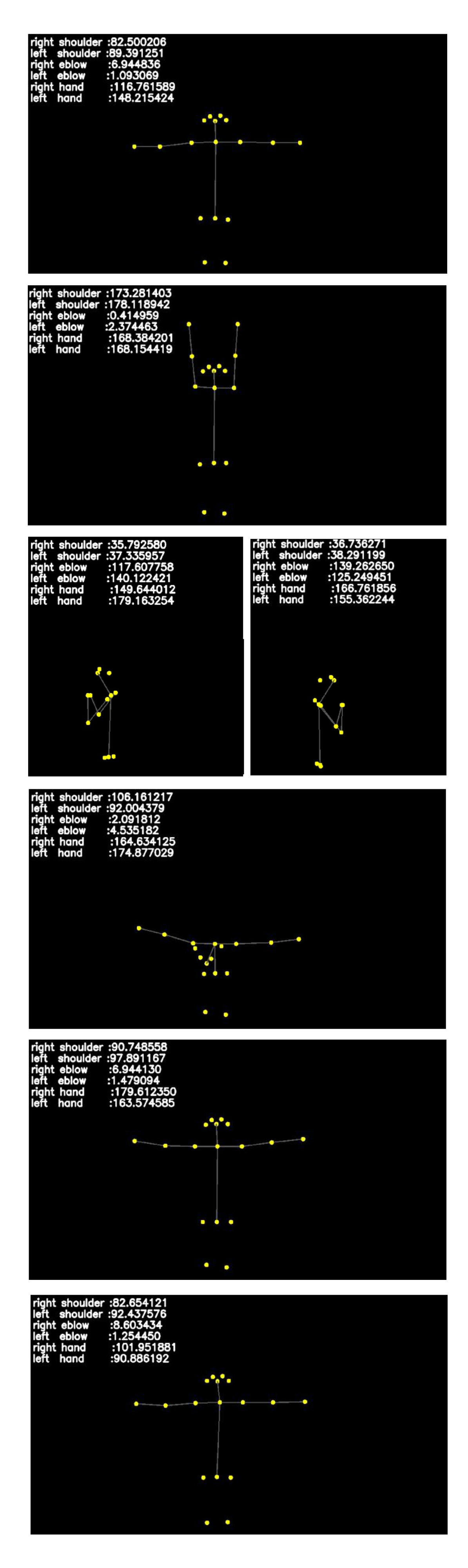

Supplement: Supplementary file 2 — Additional file 2: Supplementary Figure 2. Results obtained from the proposed method. [file 12891_2022_5826_MOESM2_ESM.tif]
